# The Effect of Free Androgen Index on the Quality of Life of Women With Polycystic Ovary Syndrome: A Cross-Sectional Study

**DOI:** 10.3389/fphys.2021.652559

**Published:** 2021-05-24

**Authors:** Mohammed Altigani Abdalla, Harshal Deshmukh, Irfaan Mohammed, Stephen Atkin, Marie Reid, Thozhukat Sathyapalan

**Affiliations:** ^1^Department of Academic Diabetes, Endocrinology, and Metabolism, Hull York Medical School, University of Hull, Hull, United Kingdom; ^2^School of Postgraduate Studies and Research, RCSI Medical University of Bahrain, Muharraq, Bahrain; ^3^Department of Psychology, Faculty of Health Sciences, University of Hull, Hull, United Kingdom

**Keywords:** polycystic ovary syndrome - quality of life - modified PCOS questionnaire - anti-Mullerian hormone - free androgen index, PCOS, psychology, quality of life, reproductive endocrine

## Abstract

**Purpose:** Free androgen index (FAI) and anti-Mullerian hormone (AMH) are independently associated with polycystic ovary syndrome (PCOS). This study aimed to describe the relationship between these two markers and health-related quality of life (HR-QoL) in women with PCOS.

**Methods:** This cross-sectional study consisted of 81 women in the Hull PCOS biobank, who fulfilled the Rotterdam consensus criteria for the diagnosis of PCOS. The primary outcome was to measure the various domains of the QoL in the modified polycystic ovary syndrome questionnaire (MPCOSQ).

**Results:** Mean age of the study participants was 28 ± 6.0 years, mean body mass index (BMI) 33.5 ± 7.8 kg/m^2^, mean FAI (6 ± 5.5), free testosterone (2.99 ± 0.75) and mean AMH (3.5 ± 0.8 units). In linear regression analysis, the FAI was associated with overall mean MPCOSQ score (Beta = 0.53, *P*-value = 0.0002), and with depression (Beta = 0.45, *P*-value = 0.01), hirsutism (Beta = 0.99, *P*-value = 0.0002) and menstrual irregularity (Beta = 0.31, *P*-value = 0.04). However, with adjustment for age and BMI, FAI was only associated with the hirsutism domain (Beta = 0.94, *P*-value = 0.001) of the MPCOSQ. FAI was also associated with the weight domain (Beta = 0.63 *P*-value = 0.005) of MPCOSQ. However, AMH was not associated with the overall mean MPCOSQ score or with any of its domains.

**Conclusion:** FAI but not AMH was associated with QoL in women with PCOS, and this effect was mediated by BMI.

## Introduction

Polycystic ovary syndrome (PCOS) affects approximately 20% of women of reproductive age (Barnard et al., 2007). It is a heterogeneous endocrine disorder associated with biochemical and clinical manifestations of hyperandrogenism, polycystic ovarian morphology, and ovulatory dysfunction (Podfigurna-Stopa et al., 2015; Amiri et al., 2019). Reproductive issues are common in women with PCOS whose presenting symptoms varying from an increased level of subfertility to an early loss of pregnancy (Homburg, 2006). Over 90% of women visiting fertility clinics with failure to conceive have PCOS (Barnard et al., 2007). Other adverse complications include menstrual irregularities, increased body weight, insulin resistance, increased risk of diabetes and cardiovascular disorders (Kamalanathan et al., 2013). The spectrum of PCOS symptoms such as acne, hirsutism, obesity, infertility, and loss of femininity imposes a significant impact on the quality of life in women with PCOS (Barnard et al., 2007).

Moreover, these symptoms are the main drive for depression, anxiety, mood swings, sexual problems, social maladaptation, and other psychological disabilities (Barnard et al., 2007; Podfigurna-Stopa et al., 2015). These factors collectively contribute to a significant reduction in the quality of life (QoL) in women with PCOS. A reduced QoL is associated with lower levels of productivity, self-esteem, and satisfaction, as well as complexities in effective weight management, aggressive behaviors, menstrual irregularities, and infertility (Acmaz et al., 2013). The prevalence of depression among women with PCOS is much higher than in the general population varying from 30 to 65% (Podfigurna-Stopa et al., 2015). Common feelings of women with PCOS include sadness, frustration, fear of not having children, restlessness, anxiety, feeling unattractive, headaches, and feeling worried and embarrassed (Acmaz et al., 2013). Other common symptoms of depression include feeling tense, loss of appetite, increased level of distraction, and loss of concentration (Ching et al., 2007). Anxiety is another common problem in women with PCOS, ranging from 35 to 56% (Podfigurna-Stopa et al., 2015). Menstrual irregularities, hirsutism, acne, infertility, and weight problems are the most distressing symptoms for women with PCOS (Trent et al., 2003). However, there is uncertainty regarding which features of PCOS play a pivotal role in depression and hence low QoL. One study found that high body mass index (BMI) is the primary factor for reducing health-related Quality of life (HR-QoL) in young women with PCOS (Trent et al., 2005). Another study reported obesity in women with PCOS with a BMI greater than 30 kg/m^2^ to be related to lower resistance to stress, raised negative emotion and low self-esteem (Komarowska et al., 2013). Testosterone in the other hand, plays a pivotal role in the pathophysiology of PCOS (Koch, 2011). It has been found that a level of testosterone even slightly outside the normal range for females is linked to the most severe type of depression and aggression (Hahn et al., 2005). At the same time, women with PCOS taking oral contraceptives (OC) as part of their treatment experienced a milder form of depression due to lower androgen levels compared to those not on OC (Rasgon et al., 2003).

We (Sathyapalan et al., 2018; Deshmukh et al., 2019) and others (Abbara et al., 2019; Calzada et al., 2019; Evliyaoglu et al., 2020) have recently reported anti-Mullerian hormone (AMH) as an independent predictor of PCOS. AMH is a surrogate marker for the follicle count and has emerged as an alternative to ultrasound scan in women with PCOS. Higher levels of AMH seen in women with PCOS can mean more severe disease and can contribute to symptoms and poorer QoL in women with PCOS however, the effect of AMH on QoL in women with PCOS is unknown. There is also an emerging evidence suggesting that insulin resistance, a common feature in PCOS, is associated with a specific pattern of PCOS in clinical practice (Hassa et al., 2006; Alviggi et al., 2017).

Thus, it is essential to understand the hormonal parameters that predict QoL in women with PCOS, as this can identify patients with PCOS who are at risk of depression and poorer QoL. The objective of this study was to examine the effect of high androgen level (FAI) and AMH on QoL in women with PCOS.

## Materials and Methods

### Design

This was a cross-sectional study involving 81 women (all ethnically identified as White) with PCOS who presented sequentially and prospectively at the Department of Academic Diabetes, Endocrinology and Metabolism between the years of 2012 and 2018, were recruited to the PCOS biobank, and had completed the modified PCOS questionnaire (MPCOSQ). The diagnosis of PCOS was based on at least two out of three of the diagnostic criteria of the Rotterdam consensus 2003 (Rotterdam Ea-SPcwg, 2004), namely clinical and biochemical evidence of hyperandrogenism [Ferriman-Gallwey score > 8; free androgen index (FAI) > 4, total testosterone > 1.5 nmol/L], oligomenorrhea or amenorrhea and polycystic ovaries on transvaginal ultrasound. Non-classical 21-hydroxylase deficiency, hyperprolactinemia, Cushing’s disease, and androgen-secreting tumors were excluded.

### Ethical Consideration

The study was approved by the Newcastle and North Tyneside Ethics committee (ISRCTN70196169) and was conducted as per the Declaration of Helsinki and local regulations. Written informed consent was obtained, following which the participants completed the MPCOSQ including validated measurement of the QoL. A separate form was used to capture the demographic characteristics of the participants including age.

### Study Measurements

MPCOSQ: We used the modified PCOSQ for this study which contained 30 items, including the acne subscale, and scales for body image and depression (Table 2). The questionnaire was administered by primary investigators for the study and handed out to the patients during their visit to the PCOS clinic. It covers a series of questions where all responses were captured on a 7-point Likert-type scale from 1 being (strongly disagree) to 7 (strongly agree). The MPCOSQ has been showed to have an acceptable and significant validity, consistency, and reliability (Taghavi et al., 2015).

### Laboratory Measurement

All the laboratory measurements have been detailed previously (Deshmukh et al., 2019). Briefly, blood samples were centrifuged within 5 min of collection and were stored frozen at −80°C pending analysis. All study measurements and analysis were performed following the relevant guidelines and regulations. Serum testosterone was measured by LC/MS/MS on an Acquity UPLC system coupled to a Quattro Premier XE mass spectrometer (Waters, Manchester, United Kingdom). We achieved a lower limit of quantification of 5 pmol/L for testosterone and 0.01 ng/mL for AMH. Recovery at 83–116% for the analyte, and the assay was found to be linear up to 2,500 pmol/L for testosterone. Furthermore, the inter-assay precision and bias were calculated at concentrations of 25, 50, and 100 pmol/L for each analyte. The cumulative variance (CV) was found < 4% for testosterone (bias < 4%). Sex hormone-binding globulin (SHBG) was measured by an immunometric assay with fluorescence detection on the DPC IMMULITE 2000 analyzer using the manufacturer’s recommended protocol (upper limit of the reference range 2.0 nmol/L). The FAI was calculated as the total testosterone × 100/SHBG. Anti-Müllerian hormone was measured using a Beckman Coulter Access automated immunoassay.

### Statistical Analysis

All data were analyzed using R 3.6.1. [R Core Team. R: A Language and Environment for Statistical Computing (Internet), Vienna, Austria; 2016]. Available from: https://www.R-project.org/. Means, standard deviations (SD), medians, interquartile ranges (IQR) and the means for MPCOSQ domains were computed. All values are shown as mean ± SD unless otherwise specified. Significance was defined as *p* ≤ 0.05. Linear regression analysis was used to determine the effect of FAI and AMH on the overall mean score of the MPCOSQ and its sub-domains with adjustments to age and BMI.

## Results

The baseline characteristics of participants are summarized as mean ± SD for the continuous variables. Overall, the participants were relatively young adults (mean age 28 ± 6 years) and tend to be overweight or obese (mean BMI 33.5 ± 7.8 kg/m^2^); mean weight (93.6 ± 22.8 kg); with elevated levels of markers for hyperandrogenaemia (FAI 6 ± 5.5), total testosterone (1.7 ± 1 nmol/L) and the free testosterone (2.99 ± 0.75) (Table 1). The different domains of the MPCOSQ with the participant’s responses (Mean ± SD) are summarized in Table 2. In the linear regression analysis, FAI was significantly associated with total MPCOSQ scores (Beta = 0.53, *P*-value = 0.0002) and with its subdomains including depression (Beta = 0.45, *P*-value = 0.01), hirsutism (Beta = 0.99, *P*-value = 0.0002) and menstrual irregularity (Beta = 0.31, *P*-value = 0.04). However, after adjusting for age and BMI, FAI was only significantly associated with hirsutism (Beta = 0.94, *P*-value = 0.001). Moreover, FAI was also associated with the MPCOSQ weight domain (Beta = 0.63, *P*-value = 0.005). On the other hand, no association was established between AMH and total MPCOSQ score or any of its sub-domains (Table 3).

**TABLE 1:** Baseline characteristics of the study population.

**Parameters**	**Mean ± SD**
Age (years)	286
BMI (kg/cm^2^)	33.57.8
Weight (kg)	93.622.8
Baseline glucose (nmol/L)	4.70.5
2 h Glucose (nmol/L)	5.91.7
Triglyceride (mmol/L)	1.41
HDL (mmol/L)	1.40.3
LDL (mmol/L)	2.50.6
Cholesterol (mmol/L)	4.81
CRP (mg/L)	4.24
Androstenedione (nmol/L)	10.55`
Insulin (Ulu/L)	117.7
FAI	65.5
AMH (pmol/L)	3.50.8
Testosterone (nmol/L)	1.71
Free testosterone (pmol/L)	2.990.75
DHEAS (umol/L)	5.93
17-OHP (nmol/L)	5.83
SHBG (nmol/L)	3.30.5
Estradiol (pmol/L)	234161
LH (iu/L)	6.13.5
FSH (iu/L)	4.52.3

BMI, body mass index; HDL, high-density lipoprotein; LDL, low-density lipoprotein; CRP, c-reactive protein; FAI, free androgen index; AMH, anti-Mullerian hormone; DHEAS, dehydroepiandrosterone; 17-OHP,17α- hydroxyprogesterone; LH, luteinizing hormone; FSH, Follicular stimulating hormone. ^*^Free testosterone was obtained using Vermeulen formula.

**TABLE 2:** The overall Mean scores of six domains of the MPCOSQ.

**Domains/items**	**Means ± SD**
**Body Hair**	
Growth of visible hair on the chin	3.22.2
Growth of visible hair on upper lip	3.11.9
Growth of visible hair on your face	2.72.1
Growth of visible body hair	3.21.8
Embarrassed about excessive body hair	3.52.2
**Depression**	
Depressed as a result of having PCOS	3.02.0
Moody as a result of having PCOS	3.41.7
Dealing with the diagnosis of PCOS	4.31.5
Have low self-esteem as a result of PCOS	3.31.9
Worried about having PCOS	3.11.9
Self-conscious as a result of having PCOS	3.61.8
**Menstrual symptoms and menstrual predictability**	
Irregular period	42.0
Menstrual cramp	3.91.7
Headache	3.31.8
Acne with menstrual period	1.91.9
Abdominal bloating	3.81.7
Late period	3.52.0
**Acne**	
Unattractive as a result of acne	1.91.7
Depressed as a result of having acne	1.81.3
**Infertility**	
Concerned about infertility	2.82.5
Afraid not being able to have kids	2.92.5
Feel sad because of infertility	2.92.3
**Weight**	
Concerned about being overweight	4.31.8
Felt frustrated trying to lose weight	4.91.4
Having difficulties trying to stay at ideal weight	5.21.3
Feel not attractive because of being overweight	4.71.8
Had trouble dealing with your weight	4.71.7

*SD, standard deviation; PCOS, polycystic ovary syndrome; MPCOSQ, modified PCOS questionnaire.*

**TABLE 3:** Regression analysis for the effect of FAI and AMH on the domains of the MPCOSQ.

**Domains**	**Free androgen index (FAI)**				**Anti-Mullerian hormone (AMH)**			
	Beta (SE)^*^	***P*-value**	Beta (SE)^**^	***P*-value**	Beta (SE)^*^	***P*-value**	Beta (SE)^**^	***P*-value**
Depression	0.45 (±0.18)	0.01	0.07 (±0.18)	0.68	−0.16 (±0.16)	0.33	−0.17 (±0.15)	0.26
Weight¥	0.63 (±0.22)	0.005	0.63 (±0.22)	0.005	0.63 (±0.22)	0.005	0.03 (±0.2)	0.87
Hirsutism	0.99 (±0.25)	0.0002	0.94 (0.29)	0.001	−0.25 (±0.23)	0.28	−0.25 (±0.23)	0.28
Acne	0.10 (±0.21)	0.62	−0.03 (±0.24)	0.87	−0.14 (±0.19	0.45	−0.15 (±0.19)	0.44
Menstrual irregularities	0.31 (±0.15)	0.04	0.2 (±0.17)	0.23	0.04 (±0.13)	0.72	0.04 (±0.13)	0.74
Infertility	0.58 (±0.31)	0.06	0.1 (±0.33)	0.76	−0.04 (±0.28)	0.86	−0.05 (±0.26)	0.83
Overall	0.53 (±0.13)	0.0002	0.26 (±0.14)	0.06	−0.08 (±0.12)	0.49	−0.09 (±0.11)	0.43

^*^Adjusted for age, ^**^Adjusted for age and BMI, and ¥Adjusted to weight; FAI, free androgen index; AMH, anti-Mullerian hormone; MPCOSQ, modified PCOS questionnaire; SE, standard error. *P*-value: significant at <0.05.

## Discussion

In this study we assessed the effect of FAI and AMH on quality of life and psychological well-being amongst women with PCOS. We found that FAI but not AMH was associated with quality of life in women with PCOS and that this effect was largely mediated by FAI’s effect on body weight and BMI. FAI was associated with total MPCOSQ along with several subscales of MPCOSQ including depression, weight, hirsutism, and menstrual irregularities. However, adjustment for BMI and weight attenuated those associations. The findings of our study agree with earlier studies looking at the effect of hyperandrogenism on QoL (Livadas et al., 2011; Dokras, 2012). Moreover, FAI was strongly associated with the weight subscale, suggesting that its impact on BMI mediates the effects of FAI on the QoL in women with PCOS. Hyperandrogenaemia seems to be the only feature consistently associated with metabolic disturbances in women with PCOS (Pehlivanov and Orbetzova, 2007; Guastella et al., 2010; Guo et al., 2010; Yilmaz et al., 2011; Panidis et al., 2012), and here we showed that its association with QoL was mediated by its effect on weight/BMI in women with PCOS. It has been shown that both weight loss and lowering the androgen levels resulted in significant improvements in both the physical and the mental aspects of the QoL of women with PCOS (Dokras et al., 2016).

There are some data showing that psychological stress is related to a decreased AMH level in infertile women with PCOS and this psychological stress may affect the ovarian reserve (Dong et al., 2017). However, there are no studies looking at AMH levels and its association with QoL in women with PCOS who usually have higher levels of AMH (Sathyapalan et al., 2018; Deshmukh et al., 2019). Therefore, in this study we showed that AMH levels were not associated with QoL in women with PCOS.

### Strength and Limitations

We used the MPCOSQ to assess quality of life in women with PCOS. The MPCOSQ has six subscales, its reliability was studied previously, and its internal consistency was found to be acceptable (Barnard et al., 2007; Bazarganipour et al., 2012). Moreover, the validity of its content has also been assessed and is considered very satisfactory (Bazarganipour et al., 2012). Factor analysis has been used to assess the structural validity of MPCOSQ and generally consist of 5–6 domains (Elsenbruch et al., 2003; Guyatt et al., 2004; Barnard et al., 2007). In this study, we used MPCOSQ with six domains, same as what previously reported by Barnard et al. (2007). The lack of an acne scale in the previous MPCOSQ has been a point of criticism as many studies underestimated the influence of acne on the QoL (Jones et al., 2004; Rall et al., 2014); therefore, we added an acne domain to the MPCOSQ used in this study.

The study has several limitations. One of the limitations of our study is the small sample size and hence limited power to estimate the effect of FAI on QOL. However, we note that this is the first study looking at the effect of FAI on QOL and there are limited data in the literature to do an informed power calculations. Therefore, a well-designed clinical trials are needed to provide a true insight into the significant effect of the FAI on the QoL of women with PCOS. Furthermore, it was a small cross-sectional study, so causality cannot be ascertained. Similarly, women with PCOS with predominant weight and hirsutism concerns may be referred to endocrinology clinic, whereas those with predominantly interfertility concerns are referred to the gynecology clinics. Hence there may be an underestimation of infertility concerns and its association with FAI and AMH. The study also lacks the demographic and social characterizations such as the marital status, money income and level of education. Moreover, psychological characterization including previous history of anxiety and depression was not covered by questionnaire. However, despite these limitations, our study had several advantages. It includes cohort of women with PCOS, and we report the association of the QoL with both FAI and AMH. To the best of our knowledge, no studies have looked at the association of the QoL in women with PCOS with AMH levels.

In summary, we report that FAI and not AMH influence the QoL in women with PCOS through its effect on weight and BMI. Therefore, it seems likely that effective weight loss strategies including dietary interventions, exercise, pharmacological interventions, and educational programs can improve QoL of women with PCOS.

## Data Availability Statement

The raw data supporting the conclusions of this article will be made available by the authors, without undue reservation.

## Ethics Statement

The studies involving human participants were reviewed and approved by Newcastle North Tyneside Ethics committee (ISRCTN70196169). The patients/participants provided their written informed consent to participate in this study. All parties consented to the publication of the study.

## Author Contributions

MA conceived the presenting idea, developed the theory, and wrote the manuscript with support from IM, SA, MR, and TS. HD performed the computations. All authors contributed to the article and approved the submitted version.

## Conflict of Interest

The authors declare that the research was conducted in the absence of any commercial or financial relationships that could be construed as a potential conflict of interest.
